# Improvement in survival among HIV-infected individuals in the Republic of Korea: Need for an early HIV diagnosis

**DOI:** 10.1186/1471-2334-9-128

**Published:** 2009-08-12

**Authors:** Mee-Kyung Kee, Jin-Hee Lee, Eun-Jin Kim, Jiae Lee, Jeong-Gu Nam, Byung-Hee Yoo, Sung Soon Kim

**Affiliations:** 1 Division of AIDS, Korea National Institute of Health, Korea Centers for Disease Control and Prevention, Seoul, Korea; 2 Division of HIV and TB Control, Korea Centers for Disease Control and Prevention, Seoul, Korea

## Abstract

**Background:**

There is little information describing survival in HIV-infected patients after primary diagnosis in Korea, and changes in survival over time. This study investigated survival times, survival characteristics, and changes in survival after initial HIV diagnosis. Survival was characterized by evaluation of the immune status at primary HIV diagnosis nationwide.

**Methods:**

A total of 5,323 HIV-infected individuals were registered with the government and followed until the end of 2007. Survival following HIV diagnosis was estimated based on epidemiological characteristics. We examined 3,369 individuals with available initial CD4+ T-cell counts within 6 months of HIV diagnosis to estimate survival based on immune status at diagnosis. The association between epidemiological variables and survival times was analyzed with univariate and multivariate Cox's proportional hazards model.

**Results:**

Individuals died during the study period (n = 980), and 45% of the individuals died within 6 months of HIV diagnosis. The median survival following HIV diagnosis was 16.7 years. Survival were longer in women, in younger persons, in individuals diagnosed at blood centers, and in individuals diagnosed later in the study period. Survival were shortest in individuals with CD4+ T-cell counts <200 cells/mm^3 ^at HIV diagnosis. These results suggest that early HIV diagnosis in Korea is imperative to increase survival and to promote the quality of life for HIV-infected individuals with governmental support.

**Conclusion:**

The median survival time of HIV-infected individuals following HIV diagnosis was 16.7 years in Korea. The survival was significantly lower in individuals with CD4+ T-cell counts <200 cells/mm^3 ^at HIV diagnosis and higher by introduction of drugs and development of therapy.

## Background

There are nearly 60 million individuals infected with Human Immunodeficiency Virus (HIV) since the early 1980s, and at least 25 million HIV-infected individuals have died of Acquired Immunodeficiency Syndrome (AIDS) since the beginning of the epidemic [1]. AIDS has decreased life expectancies by 20 years, and the high rates of HIV infection in adolescents and women of reproductive age have resulted in community destruction, family dissolution, and economic loss in many sub-Saharan African countries. AIDS prevention program has been actively performed, and there has been significant ongoing research focused on the development of an HIV vaccine and effective antiretroviral drugs. As a result, the dead by AIDS has decreased from 2.2 million in 2005 to 2 million in 2007 and newly HIV-infected individuals have also reduced recently [1]. AIDS-associated mortality increased by 16% year by year to the mid-1990s and then was raised as first cause of adult death, however, it has rapidly decreased since the mid-1990s in the United States [2]. This decrease in AIDS-associated mortality coincided with the introduction of highly active antiretroviral therapy (HAART), including protease inhibitors [3], suggesting that survival following HIV infection increased from 10–12 years to 25 years [4,5].

A total of 5,323 individuals have been diagnosed with HIV infection between 1985 and 2007 in Korea. Approximately 91% of infected individuals were male and 99% were infected by sexual contact [6]. HIV-infected individuals may refuse antiretroviral therapy because of efficacy concerns and social stigmas. Additionally, patients and their families may remain unconvinced by positive antiretroviral effects on survival times [7]. There are no current estimates describing the survival of HIV-infected individuals in Korea, and we therefore assumed that HIV survival times might have increased with the advent of governmental support for therapy and the introduction of HAART in 1997. The number of newly diagnosed HIV-infected individuals in Korea and the target population for therapy is increasing, and it is therefore necessary to assess the national HIV/AIDS prevention program of therapy for HIV-infected individuals. It is therefore necessary to estimate HIV/AIDS survival in Korea in order to predict governmental budgets for therapy.

It is difficult to predict the time of HIV infection in this population since the majority of reported HIV infections in Korea are the result of sexual contact. This study investigated survival times, survival characteristics, and the changes in survival times following HIV diagnosis. This study also evaluated the immune status of individuals at HIV diagnosis to elucidate the association between survival and CD4+ T-cell counts at primary HIV diagnosis.

## Methods

### HIV diagnosis and the National control of HIV/AIDS

HIV-reactive samples from screening testing centers were referred to local Institutions of Health and the Environment (IHEs) or the Division of AIDS at the Korea Centers for Disease Control and Prevention (KCDC) to confirm HIV infection. Results of HIV infection were transferred to the Division of HIV & TB Control at the KCDC. Individuals were automatically registered as infected unless they were anonymously tested. Epidemiological investigations, public health education programs, connection to medical center, and therapy costs were supported by Public Health Centers (PHCs). HIV-associated deaths were documented by PHCs, and medical certificates with the dates, places, and reasons of death were reported to the Division of HIV & TB Control in KCDC. Additionally, immune testing of registered individuals was performed on a regular basis by the Division of AIDS at the KCDC or by medical centers [6].

### Subjects

A total of 5,323 HIV-infected individuals were registered at the KCDC from 1985 to 2007 in Korea. Data from these individuals were investigated to estimate survival and their association with epidemiologic characteristics following HIV diagnosis. Survival according to immune status at HIV diagnosis were estimated in individuals with initial CD4+ T-cell counts at HIV diagnosis (n = 3,369). They were older than 14 years of age at HIV diagnosis and had available initial CD4+ T-cell counts within 6 months of HIV diagnosis including individuals died of AIDS within 6 months after HIV diagnosis.

### Selection criteria

The date of HIV diagnosis was defined as the date when the KCDC or local IHEs confirmed a sample as HIV-positive. Survivals were analyzed with epidemiological variables including gender, age, screening site, year of diagnosis, and immune status at HIV diagnosis. Screening sites were classified into three groups: public institutions, hospitals, and blood centers [8]. Year of diagnosis were grouped by changes in drug introduction and therapeutic administration. Zidovudine (AZT) monotherapy was introduced to the Korean HIV therapeutic system in 1991 [9], and HAART was introduced in 1997 [10]. HIV RNA quantitative assays have been performed at the Division of AIDS at the KCDC or in medical centers to monitor the efficacy of therapy since late 2002. Categories included: no antiretroviral therapy (1985–1990), AZT monotherapy (1991–1996), HAART (1997–2002), and use of HIV RNA quantitative tests for monitoring (2003–2007). The immune status of infected individuals was stratified into four categories by CD4+ T-cell counts (<200 cells/mm^3^, 200–349 cells/mm^3^, 350–499 cells/mm^3^, and ≥500 cells/mm^3^) based on the 1993 revised classification system for HIV infection [11] and the 2002 guidelines for the use of antiretroviral agents in HIV-infected adults and adolescents by Centers for Disease Control and Prevention of United States [12].

### Statistical analysis

Follow-up of HIV-infected individuals for survival analysis of 5,323 individual was performed until December 32, 2007. Kaplan-Meier analysis and log-rank tests were used to compare survival curves stratified by epidemiological group. The Cox's proportional hazards model was applied to examine the association between variables and survival times. We classified 1,954 individuals without CD4+ T-cell counts or CD4+ T-cell counts taken greater than 6 months after HIV diagnosis into the "missing group" in the multivariate analysis to adjust the confounding. The results of the missing group were not shown in Table 1. Analysis was performed using the SAS version 9.1 software packages, and the STATISTICA program was used for survival graph.

**Table 1:** Characteristics of the study population, 1985–2007

Categories	All (%)	No. of deaths (%)	No. of deaths within 6 months of diagnosis(%)*	Person-years	Mortality density (per 1,000 person-years)
Total individuals	5,323	980 (18)	441 (45)	21,439	45.7
Gender					
Men	4,861 (91)	901 (19)	413 (46)	19,082	47.2
Women	462 (9)	79 (17)	29 (37)	2,357	33.5
Age					
≤ 29	1,363 (26)	148 (11)	25 (17)	7,153	20.7
30–39	1,758 (33)	283 (16)	102 (36)	7,506	37.7
40–49	1,220 (23)	268 (22)	136 (51)	4,081	65.7
≥ 50	982 (18)	281 (29)	179 (64)	2,699	104.1
Screening site					
Public institution	1,394 (26)	296 (21)	51 (14)	8,031	36.8
Hospital	3,448 (65)	644 (19)	390 (56)	10,632	60.6
Blood center	481 (9)	40 (8)	1 (9)	2,776	14.4
Year of diagnosis					
1985–1990	124 (2)	86 (69)	5 (6)	1,403	61.3
1991–1996	497 (10)	215 (43)	35 (16)	4,878	44.1
1997–2002	1,388 (26)	339 (24)	146 (43)	8,202	41.3
2003–2007	3,314 (62)	340 (10)	256 (75)	6,956	48.9

*%; proportion of the number of the deaths within 6 months after HIV diagnosis divided by the number of deaths

## Results

### Epidemiological characteristics of study populations

The majority (91%) of HIV-infected individuals (n = 4,861) were men (Table 1). The greatest proportion of HIV-infected individuals were aged 30–39 years (33%), and 65% of all infections were identified in hospitals. Additionally, 62% were diagnosed between 2003 and 2007 and 18% (n = 980) died before the end of the study. Additionally, 45% of individuals died within 6 months of HIV diagnosis, and many of these patients were aged 40 years and older, were initially detected in hospitals (56%), and were diagnosed between 2003 and 2007 (75%).

**Table 2:** Proportion of survival following HIV diagnosis and the estimated hazard ratios for variables

			Unadjusted		Adjusted	
			
Variable	No. of cases	Proportion surviving after 10 years (95% CI)	Hazard Ratio (95% CI)	p-value	Hazard Ratio (95% CI)	p-value
Gender						
Men	4,861	67%(64–69)	1.31(1.04,1.65)	0.0211	1.37(0.09,1.73)	0.0082
Women	462	73%(66–78)	1.00	-	1.00	-
Age						
≤ 29	1,363	81%(77–84)	1.00	-	1.00	-
30–39	1,758	71%(66–74)	1.80(1.47,2.20)	< .0001	1.46(1.19,1.80)	0.0003
40–49	1,220	56%(50–63)	2.94(2.40,3.61)	< .0001	2.10(1.70,2.60)	< .0001
≥ 50	982	49%(41–57)	4.37(3.56,5.35)	< .0001	3.08(2.48,3.83)	< .0001
Screening site						
Public institution	1,394	68%(64–71)	1.00	-	1.00	-
Hospital	3,448	68%(64–72)	1.47(1.27,1.70)	< .0001	1.50(1.27,1.76)	< .0001
Blood center	481	85%(78–89)	0.39(0.28,0.55)	< .0001	0.62(0.44,0.88)	0.0067
Year of diagnosis						
1985–1990	124	56%(46–64)	2.15(1.65,2.80)	< .0001	5.03(3.77,6.71)	< .0001
1991–1996	497	62%(57–66)	1.57(1.29,1.90)	< .0001	3.48(2.81,4.30)	< .0001
1997–2002	1,338	70%(66–73)	1.31(1.11,1.54)	0.0011	1.42(1.21,1.68)	< .0001
2003–2007	3,314	-	1.00	-	1.00	-
CD4+ T-cell counts*						
<200	1,169	46%(41–51)	5.51(4.47,6.78)	< .0001	5.10(4.11,6.33)	< .0001
200–349	868	70%(63–75)	1.06(0.81,1.38)	0.6872	1.13(0.86,1.47)	0.3865
350–499	667	80%(74–84)	0.77(1.81,2.92)	0.0757	0.80(0.60,1.06)	0.1223
≥500	665	80%(75–84)	1.00	-	1.00	-

CI: Confidence Interval

*Individuals were classified by initial CD4+ T-cell counts within 6 months of HIV diagnosis, including individuals who died from AIDS within 6 months of HIV diagnosis. Individuals without CD4+ T-cell counts or CD4+ T-cell counts measured greater than 6 months of HIV diagnosis were designated as the "missing group" (data not shown).

### The survival by epidemiologic characteristics and immune status at HIV diagnosis

**Figure 1:**
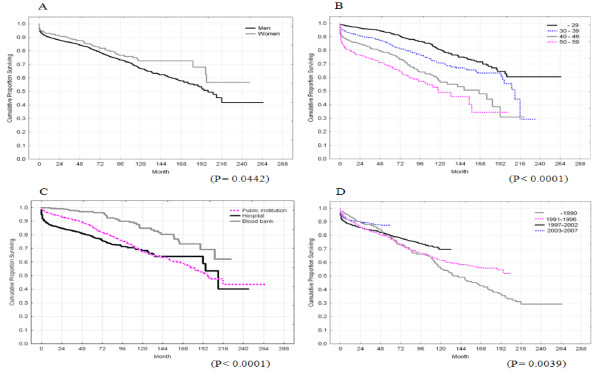
**Survival after HIV diagnosis stratified by epidemiologic characteristics**. A, Gender; B, Age; C, Screening site; and D, Year of diagnosis.

The median survival of HIV-infected individuals following diagnosis was 16.7 (data not shown). Figure 1 showed survival of HIV-infected individuals by epidemiological characteristics. Women had longer survival than men (p = 0.0442), a younger age at initial HIV diagnosis was associated with a significantly longer survival (p < 0.0001). HIV-infected individuals identified in blood centers demonstrated the longest survival, followed by individuals from public institutions and hospitals (p < 0.0001). Individuals diagnosed between 2003 and 2007 demonstrated longer survival (p = 0.0039).

**Figure 2:**
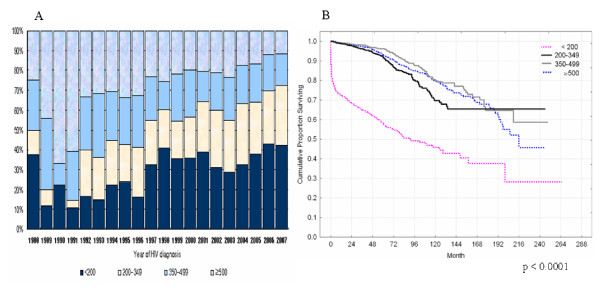
**A, Level of CD4+ T-cell counts at HIV diagnosis over calendar year, and B, Survival stratified by CD4+ T-cell counts at HIV diagnosis (The survival curve of "missing group" was not shown)**.

Survivals stratified by CD4+ T-cell counts at HIV diagnosis were shown in Figure 2. HIV-infected individuals with CD4+ T-cell counts <200 cells/mm^3 ^at HIV diagnosis demonstrated 46% survival at 10 years after diagnosis. However, individuals with CD4+ T-cell counts ≥200 cells/mm^3 ^demonstrated approximately 77% survival in the same time period (data not shown).

Table 2 showed the proportion of 10 years survival after HIV diagnosis. It also analyzed estimation Cox's hazard ratio by epidemiological variables. The adjusted relative hazard ratio (AHR) by gender was approximately 1.4-fold higher in men than in women (p = 0.0082). Individuals aged 29 years or younger had lower AHRs than individuals aged 30–39 years (AHR, 1.46; p = 0.0003), and individuals aged 40–49 years (AHR, 2.10; p < 0.0001), individuals aged 50–59 years (AHR, 3.08; p < 0.0001). Survival stratified by screening site was higher in PHCs than in hospitals (AHR, 1.50; p < 0.0001), and longer survival was observed in individuals diagnosed in blood centers (AHR, 0.62; p = 0.0067). Survival were shorter in the individuals diagnosed prior to 1990 than in individuals diagnosed between 2003 and 2007 (AHR, 5.03; p < 0.0001). Survival was shorter in individuals with CD4+ T-cell counts <200 cells/mm^3 ^than in individuals with CD4+ T-cell counts ≥500 cells/mm^3 ^(AHR, 5.10; p < 0.0001). Individuals with CD4+ T-cell counts ≥200 cells/mm^3 ^displayed similar survival.

Table 3 showed the comparison of survivals by epidemiological variables in year of diagnosis. Survival in individuals aged 30–39 years and in individuals with 200–349 cells/mm^3 ^of CD4+ T-cell counts have increased after HAART.

**Table 3:** Survival following HIV diagnosis and the estimated hazard ratio of variables by year of diagnosis

		1985–1990			1991–1996			1997–2002			2003–2007	
		
Variables	Cox's Regression	Case No.(%)	HR (95%CI)	P-value	Case No.(%)	HR (95%CI)	P-value	Case No.(%)	HR(95%CI)	P-value	Case No.(%)	HR
Gender												

Men	Unadjusted	105 (85)	2.2(1.7–2.9)	< .0001	440 (89)	1.5(1.2–1.9)	< .0001	1230 (89)	1.3(1.1–1.6)	0.001	3082 (93)	1
	Adjusted		4.3(3.2–5.9)	< .0001		3.0(2.4–3.7)	< .0001		1.3(1.1–1.5)	0.0022		1

Women	Unadjusted	19 (15)	2.4(1.0–6.0)	0.0513	57 (11)	2.3(1.1–4.6)	0.0209	158 (11)	1.5(0.8–2.8)	0.2444	232 (7)	1
	Adjusted		4.3(1.6–11.4)	0.0037		4.2(2.0–9.0)	0.0001		1.6(0.9–3.1)	0.1402		1

Age												

≤ 29	Unadjusted	60 (48)	3.7(2.0–7.0)	< .0001	198 (40)	2.0(1.1–3.6)	0.0188	339 (24)	1.4(0.8–2.5)	0.2553	766 (23)	1
	Adjusted		4.8(2.5–9.3)	< .0001		2.9(1.6–5.3)	0.0007		1.5(0.9–2.8)	0.1514		1

30–39	Unadjusted	40 (32)	3.5(2.2–5.7)	< .0001	184 (37)	2.6(1.8–3.7)	< .0001	472 (34)	1.7(1.2–2.3)	0.0016	1062 (32)	1
	Adjusted		6.6(3.9–11.1)	< .0001		4.1(2.8–6.0)	< .0001		1.7(1.2–2.3)	0.0016		1

40–49	Unadjusted	18 (15)	2.3(1.3–4.1)	0.0065	76 (15)	2.5(1.7–3.6)	< .0001	323 (23)	1.4(1.1–1.9)	0.0181	803 (24)	1
	Adjusted		2.4(1.3–4.4)	0.0072		3.2(2.2–4.7)	< .0001		1.4(1.0–1.9)	0.0293		1

≥ 50	Unadjusted	6 (5)	3.7(1.6–8.3)	0.0019	39 (8)	1.5(1.0–2.4)	0.0755	254 (18)	1.2(0.9–1.5)	0.2709	683 (21)	1
	Adjusted		5.1(2.2–12.0)	0.0002		2.7(1.7–4.4)	< .0001		1.1(0.8–1.5)	0.4569		1

Screening site												

Public institution	Unadjusted	86 (69)	3.5(2.2–5.8)	< .0001	295 (59)	2.4(1.6–3.8)	0.0001	388 (28)	1.8(1.1–2.8)	0.0148	621 (19)	1
	Adjusted		4.5(2.7–7.4)	< .0001		3.1(2.0–4.9)	< .0001		1.7(1.1–2.7)	0.0248		1

Hospital	Unadjusted	17 (14)	3.7(2.1–6.5)	< .0001	121 (24)	2.8(2.1–3.7)	< .0001	857 (62)	1.6(1.3–1.8)	< .0001	2451 (74)	1
	Adjusted		4.0(2.3–7.1)	< .0001		3.5(2.7–4.7)	< .0001		1.4(1.2–1.7)	0.0003		1

Blood center	Unadjusted	21 (17)	2.5(0.5–11.3)	0.2406	81 (16)	1.5(0.3–6.1)	0.6111	143 (10)	0.7(0.2–3.1)	0.6275	242 (7)	1
	Adjusted		4.9(1.0–25.1)	0.0546		2.2(0.5–9.8)	0.3165		0.9(0.2–4.1)	0.8896		1

CD4+ T-cell counts*												

<200	Unadjusted	10 (20)	1.6(1.0–2.5)	0.0707	63 (18)	1.9(1.5–2.5)	< .0001	313 (35)	1.2(1.0–1.5)	0.0221	783 (37)	1
	Adjusted		2.7(1.6–4.4)	0.0001		2.8(2.1–3.7)	< .0001		1.3(1.1–1.6)	0.0063		1

200–349	Unadjusted	3 (6)	5.0(2.2–11.7)	0.0002	71 (21)	2.7(1.4–5.4)	0.0035	212 (24)	1.1(0.6–2.1)	0.7649	582 (28)	1
	Adjusted		6.3(2.6–15.0)	< .0001		2.7(1.4–5.5)	0.005		1.0(0.5–2.0)	0.9749		1

350–499	Unadjusted	13 (25)	1.9(0.7–5.3)	0.2351	90 (26)	1.0(0.4–2.7)	0.9422	172 (19)	0.8(0.3–2.1)	0.7298	392 (19)	1
	Adjusted		2.6(0.9–7.7)	0.081		1.1(0.4–3.0)	0.8152		0.9(0.3–2.2)	0.7546		1

≥ 500	Unadjusted	25 (49)	3.4(1.4–8.4)	0.0076	120 (35)	2.1(0.9–4.9)	0.1056	192 (22)	1.3(0.5–3.1)	0.6153	328 (16)	1
	Adjusted		3.5(1.3–9.0)	0.0117		2.2(0.9–5.4)	0.0966		1.3(0.5–3.1)	0.6118		1

CI: Confidence Interval, HR: Hazard Ratio

*Individuals were classified by initial CD4+ T-cell counts within 6 months of HIV diagnosis, including individuals who died from AIDS within 6 months of HIV diagnosis. Individuals without CD4+ T-cell counts or CD4+ T-cell counts measured greater than 6 months of HIV diagnosis were designated as the "missing group" (data not shown).

## Discussion and conclusion

The survival following HIV diagnosis in Korea was 16.7 years in this study. Survival in individuals diagnosed between 2003 and 2007 was 5.0- and 3.5-fold higher in individuals diagnosed prior to 1990 and between 1991 and 1996, respectively. This suggested the presence of positive therapeutic effects in survival of HIV-infected individuals based on introduction of government supported-antiretroviral therapy. Several hospitals were designated as AIDS care centers in 1989 during the early periods of therapeutic support in Korea [13]. However, this system was abolished due to the prohibition of discrimination against HIV-infected individuals in 1999. The number of HIV treatment hospitals increased to 60 hospitals in 2007 [14]. Survival was increased during AZT treatment periods (1991–1996) compared to non-treatment periods (1985–1990), in spite of no effect of AZT on survival in other studies [15]. This may be the result of increased immunity due to dietary health support products such as Korean red ginseng, increased support for HIV-infected individuals, and rapid contact with hospitals rather than due to therapeutic effect of AZT [16,17]. During HAART era, the survival was longer in 2003–2007 compared to survival in 1997–2002. This could be associated with appropriate HIV RNA quantitation and drug resistance testing for efficient therapy. In addition, the increased 17 available drugs compared to 15 available drugs before 2003 may have positive effect on survival in 2003–2007 [18]. The most efficient therapeutic effect in the group with CD4+ T-cell counts of 200–349 cells/mm^3 ^was due to the initiation and continued HAART in newly diagnosed individuals with CD4+ T-cell counts <350 cells/mm^3 ^according to CDC guidelines. However, HAART in newly-diagnosed individuals with CD4+ T-cell counts <200 cells/mm^3 ^did not appear efficacious.

The immune status of newly diagnosed individuals revealed low CD4+ T-cell counts, suggesting that HIV diagnosis was delayed after primary infection [8]. Individuals with CD4+ T-cell counts ≥200 cells/mm^3 ^had longer survival than individuals with CD4+ T-cell counts <200 cells/mm^3^. Survival of individuals with CD4+ T-cell counts <200 cells/mm^3 ^was increased to 13 years compared to previous results from Korea (3.6 years) and USA (2.7 years) prior to HAART [8,19].

The proportion of individuals who died within 6 months after HIV diagnosis was higher in Korea (45%) than in France (14%) or the United Kingdom (10–20%) [20,21]. Approximately 70% of individuals who died within 6 months of diagnosis were diagnosed within 2 months prior to death (data not shown). Mortality was related to age [22] and decreased CD4+ T-cell counts, suggesting that older HIV-infected individuals were generally diagnosed at the late stages of disease. This suggested that early diagnosis is required to prevent HIV transmission and allow for appropriate HIV treatment in Korea.

AIDS mortality due to HIV-related causes has been reduced by HAART. However, non-HIV-related mortality has increased [23]. AIDS-associated mortality in Korea encompassed 70% of all deaths in HIV-infected individuals, and heart disease, liver disease, cerebral disease, and respiratory and pulmonary diseases were secondary causes of death. AIDS-related mortality in HIV-infected individuals has slowly decreased, although AIDS is still the primary cause of death in Korea [20]. Non-HIV-related deaths among HIV-infected individuals included drug-associated deaths in New York (1999 to 2004), cancer in Australia and the United States (2004), hepatic disease in Europe, and hepatic disease associated with hemophilia [24-28]. However, AIDS was still the primary cause for mortality due to late diagnosis after primary infection.

There are several limitations in this study. Survivals were estimated indirectly based on changes in drug introduction and development of therapy. Drug therapy for HIV infections follows CDC guidelines in Korea [29], so grouping based on drug therapy introduction times is reasonable. However, further study is required to characterize survival based on Korean therapeutic effects, and main survival factors require the investigation of therapy acceptance or rejection, the date of antiretroviral therapy initiation, antiretroviral dosages, treatment protocols, and compliance. The study also required the investigation of survival based on smoking and alcohol consumption as primary epidemiological factors of survival. Second, survival stratified by cause of death was not analyzed in this study due to numerous AIDS-associated syndromes and subjective decisions by physicians. Additionally, cardiovascular disease, diabetes, and hepatic disease can also cause death in AIDS patients, as can myocardial infarction and metabolic complications as therapeutic side effects [30]. Therefore, the standardization of causes of death in HIV-infected individuals should analyze survival based on cause of death. Lastly, individuals without CD4+ T-cell counts or with CD4+ T-cell counts collected greater than 6 months after HIV diagnosis were designated the "missing group" during analysis. However, there were no differences between individuals with initial CD4+ T-cell counts measured within 6 months after HIV diagnosis (including individuals who died during this time period) and the "missing group" in survival stratified by immune status at diagnosis (HR: 0.94; [95% confidence interval, 0.80–1.11]; p = 0.4693).

Survival after HIV diagnosis in Korean populations has been recently increasing. A delayed diagnosis results in missed therapeutic opportunities, while an early diagnosis may increase survival and the quality of life in HIV-infected individuals supported by governmental programs. The best way to improve rates of early diagnosis in asymptomatic individuals is to test as many individuals as possible. Korean government made a stipulation for anonymous testing system in March 2008 to minimize the fear for identity exposure by HIV testing [31]. Anonymous testing services need to be improved in PHC and voluntary counseling and testing centers.

This study is the first to investigate survival of HIV-infected individuals in Korea. Future studies should evaluate survival characteristics based on disease progression to AIDS, administration of antiretroviral drugs, therapeutic protocols, and antiretroviral drug resistance.

## Competing interests

The authors declare that they have no competing interests.

## Authors' contributions

MKK participated in study design, coordination and manuscript writing. JHL participated in study design and manuscript writing and performed the statistical analysis. EJK and JL participated in coordination and manuscript writing. JGN and BHY participated in study design and acquisition of data. SSK contributed in conception, study design and coordination. All authors read and approved the final manuscript.

## Pre-publication history

The pre-publication history for this paper can be accessed here:

http://www.biomedcentral.com/1471-2334/9/128/prepub
